# Study of an Online Plan Verification Method and the Sensitivity of Plan Delivery Accuracy to Different Beam Parameter Errors in Proton and Carbon Ion Radiotherapy

**DOI:** 10.3389/fonc.2021.666141

**Published:** 2021-05-28

**Authors:** Jun Zhao, Zhi Chen, Xianwei Wu, Ying Xing, Yongqiang Li

**Affiliations:** ^1^Department of Radiation Oncology, Fudan University Shanghai Cancer Center, Shanghai, China; ^2^Department of Oncology, Shanghai Medical College, Fudan University, Shanghai, China; ^3^Shanghai Key Laboratory of Radiation Oncology, Shanghai, China; ^4^Department of Medical Physics, Shanghai Proton and Heavy Ion Center, Shanghai, China

**Keywords:** proton and carbon ion radiotherapy, scanning beam, plan verification, sensitivity, beam parameter deviation

## Abstract

For scanning beam particle therapy, the plan delivery accuracy is affected by spot size deviation, position deviation and particle number deviation. Until now, all plan verification systems available for particle therapy have been designed for pretreatment verification. The purpose of this study is to introduce a method for online plan delivery accuracy checks and to evaluate the sensitivity of plan delivery accuracy to different beam parameter errors. A program was developed using MATLAB to reconstruct doses from beam parameters recorded in log files and to compare them with the doses calculated by treatment planning system (TPS). Both carbon ion plans and proton plans were evaluated in this study. The dose reconstruction algorithm is verified by comparing the dose from the TPS with the reconstructed dose under the same beam parameters. The sensitivity of plan delivery accuracy to different beam parameter errors was analyzed by comparing the dose reconstructed from the pseudo plans that manually added errors with the original plan dose. For the validation of dose reconstruction algorithm, mean dose difference between the reconstructed dose and the plan dose were 0.70% ± 0.24% and 0.51% ± 0.25% for carbon ion beam and proton beam, respectively. According to our simulation, the delivery accuracy of the carbon ion plan is more sensitive to spot position deviation and particle number deviation, and the delivery accuracy of the proton plan is more sensitive to spot size deviation. To achieve a 90% gamma pass rate with 3 mm/3% criteria, the average spot size deviation, position deviation, particle number deviation should be within 23%, 1.9 mm, and 1.5% and 20%, 2.1 mm, and 1.6% for carbon ion beam and proton beam, respectively. In conclusion, the method that we introduced for online plan delivery verification is feasible and reliable. The sensitivity of plan delivery accuracy to different errors was clarified for our system. The methods used in this study can be easily repeated in other particle therapy centers.

## Introduction

Proton and carbon ion radiotherapy has gained great attention in recent years and has been used for different tumor sites (1–4). Compared with photon radiotherapy, proton and carbon ion radiotherapy can provide a higher target dose while allowing better sparing of normal tissue because of the depth dose distribution and a low entrance dose followed by the Bragg-peak (5–7). In addition to the physical advantage of the depth dose profile, proton and carbon ion offer differential relative biological effectiveness (RBE) with depth, which is due to a relatively low linear energy transfer (LET) in the entrance region with progressively increased LET at the peak. The physical characteristics of sharp dose peak at a particular range in tissue make the delivered dose distribution sensitive to delivery uncertainties that could make the Bragg-peak located at an incorrect position (8, 9). This may result in an insufficient dose in the target region or severe side effects. Therefore, quantitative verification is desirable to evaluate whether the treatment device delivers the correct dose distribution in each treatment fraction. Currently, all available plan verification methods were off-line and pre-treatment, using 3D pinpoint ion chamber blocks, ion chamber matrix or films measured in phantom (10–13). We cannot obtain an estimate of the delivered dose distribution during treatment with such solutions. The potential online verification methods include positron emission tomography (PET) verification and prompt gamma ray verification (14–17), which are far from fully applied in the clinical. All radiotherapy systems have log files to record the information during treatment. The log files of our radiotherapy system recorded for each treatment fraction include the information for actual delivered spot position, spot size and particle number in each spot for each iso-energy slice (IES). All log-file data that are measured by the beam monitoring system, which is in the beam nozzle in front of the patient, represents the actual beam delivery can be used to evaluate the delivered dose distribution accuracy. Moreover, for scanning beam proton and carbon ion radiotherapy, the sensitivity of plan delivery accuracy to the spot size deviation, position deviation and particle number deviation is very important for quality assurance and beam delivery system setting.

The purpose of this study is to introduce a verification method and to develop a software utilizing log file data to check the proton and carbon ion plan delivery accuracy. Moreover, the software was used to evaluate the sensitivity of plan delivery accuracy to spot size deviation, position deviation and particle number deviation.

## Methods and Materials

### Beam Delivery System

**Figure 1:**
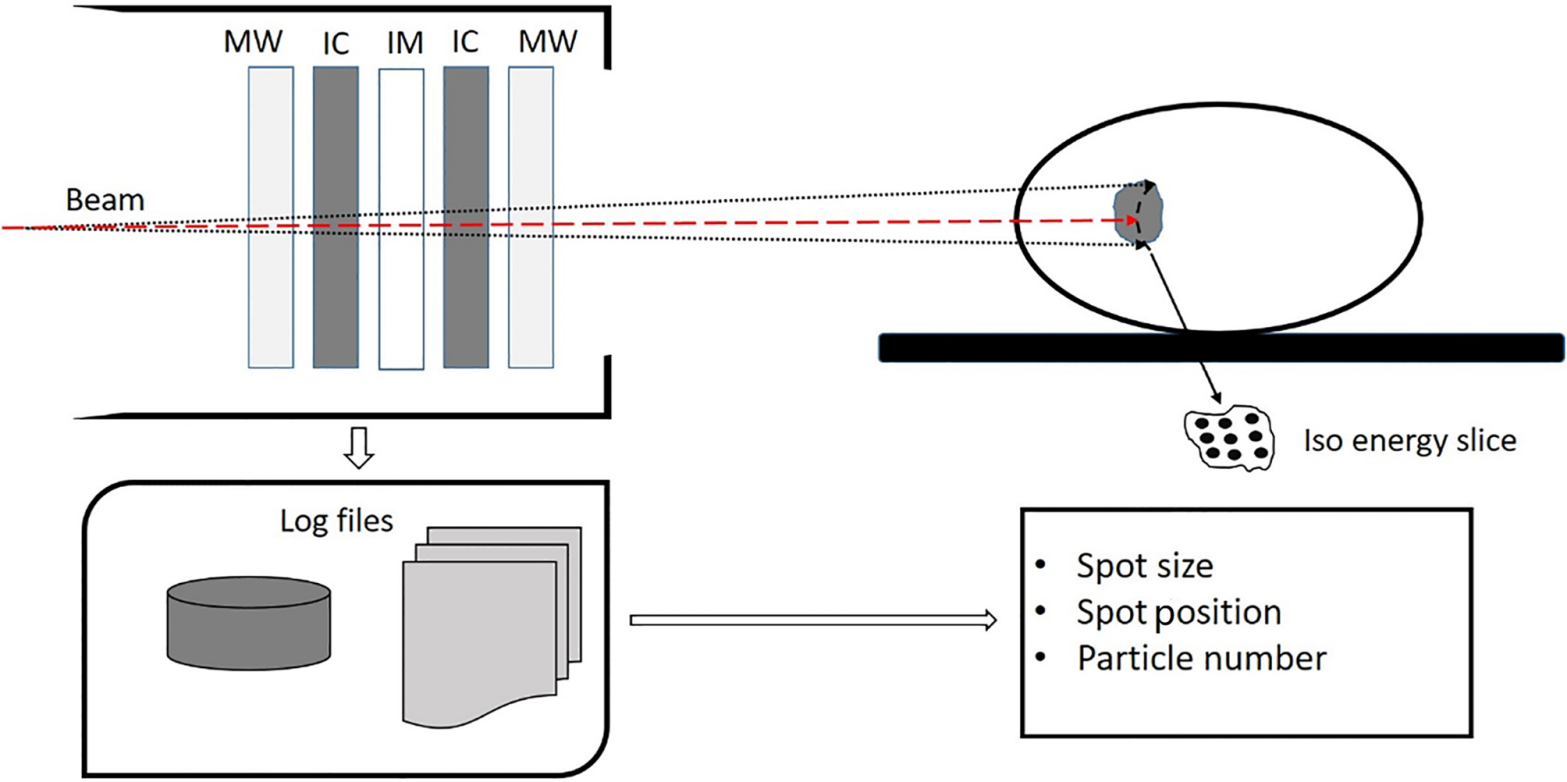
Diagram of the beam application and monitoring system (BAMS) which consists of three ion chambers (IC and IM) and two multiware proportional chambers (MW). The spot size, spot position, and particle number in each spot is measured by the BAMS and are recorded in a log file.

The Siemens ion beam delivery system used in this study has a synchrotron for acceleration and a modulated scanning beam technique paired with energy stacking for beam delivery. The accelerator can produce proton and carbon ion beams with energies ranging from 48.08 to 221.07 MeV/n and 86.22 to 430.12 MeV/n, respectively. Each ion species can be accelerated and extracted at one of the 296 different preprogrammed energy levels, which correspond to an approximately 1 mm step of range in water. For each energy level, there are five different spot sizes. The spot sizes at isocenter in air are 8.10 to 32.65 mm and 3.38 to 13.53 mm for proton and carbon ion beams, respectively. Higher energy has smaller spot size. The modulated scanning technique makes the beam remain at each spot until the requested monitor units is reached and then swiftly moves the beam to the next spot without turning off the beam. The ion beam delivery system has a dynamic intensity control system (DIC), which computes and adapts the extraction rate during the delivery of ions at each spot to make efficient and accurate extractions. Beam delivery is monitored by the beam application and monitoring system (BAMS). **Figure 1** shows the BAMS diagram. Beam parameters measured by the BAMS are recorded in the log file. The BAMS in each treatment room consists of three transmission ionization chambers and two multiwire proportional chambers. The ionization chambers are used to monitor the number of ions delivered at each spot. Multiwire proportional chambers are used to monitor the spot position and size. The spot positions and spot size (horizontal and vertical widths) are measured every 250 μs by multiwire proportional counters. The flux is sampled by a transmission parallel plate ionization chamber at 1-μs time intervals, which can quickly turn the beam to the next spot when reaching the prescribed particle numbers in one spot.

### Software Development

In our plan delivery system, the nominal plan parameters from the TPS are stored in the physical beam plan (PBP). During patient treatment, the actual plan delivery parameters are measured by the BAMS. The measured values at the BAMS position are converted to values at iso-center in air according to the base data lookup table and then stored in the physical beam record (PBR). A homemade program was developed using MATLAB to reconstruct the 2D dose distribution according to the beam parameters, such as the spot size, spot position, particle number in each spot, virtual source distance, depth dose distribution data, and double Gaussian distribution parameters. A double Gaussian distribution model is used for the lateral beam profile, which can calculate the dose at the halo area at a large radial distance more accurate than a single Gaussian distribution model (18, 19). Gamma analysis (20) was used to evaluate the difference between the reconstructed dose and plan dose to check the accuracy of treatment delivery. The plan dose is considered the reference distribution. Local normalization in the dose difference is used in gamma evaluation.

### 2D Dose Distribution Reconstruction

In this study, the dose was reconstructed in a cubic water phantom, which is also used in TPS for calculating the verification plan. Then, the dose distribution in the water phantom calculated by the TPS can be used as a reference dose to evaluate the accuracy of the reconstructed dose. The dose at a certain depth in water generated by a single pencil beam can be described as

(1) $$D=N\cdot f\left(\sigma_{1},\sigma_{2},w_{1},w_{2},x,y,z\right)\cdot DDD\left(E_{beam},z\right),$$

where *N* is the total number of delivered particles. The lateral beam profile is described by a double Gaussian distribution *f*(*σ*_1_,*σ*_2_,*w*_1_,*w*_2_,*x*,*y*,*z*), which is a the function of the beam position and depth in water. *σ*_1_, *σ*_2_, *w*_1_, and *w*_2_ are the width and weight of the first and second Gaussian distribution. The double Gaussian distribution parameters are stored in the base-data of our TPS as a lookup table as functions of depth in water for the whole required energy spectrum E. The lateral beam cutoff is 3.5 σ. *x* and *y* are the positions in x and y directions at depth *z* in water. *DDD*(*E_beam_*,*z*) is the depth dose value at depth *z* in water for the beam with energy *E_beam_*. The total 2D dose distribution in water at a certain depth is an integral of dose at the depth contributed by all beams including the fragmentation tail passing through the slice. Therefore, the 2D dose at depth *z* in water can be calculated by

(2) $$D=\int \int N_{ij}\cdot f_{ij}\left(\sigma_{1},\sigma_{2},w_{1},w_{2},x,y,z\right)\cdot {DDD}_{i}\left(E_{beam},z\right)didj,$$

where *i* is the ordinal number of IES, i.e., the beam Bragg-peak is located at *i*th IES which is related to the primary energy *E*_beam_. *j* is the ordinal number of pencil beam for each IES. *ij* represents the *j*th pencil beam for the *i*th IES. The depth dose distribution (DDD) profile and spot size (double Gaussian distribution) parameters at certain depths in water are the basic beam data in our clinical TPS.

### Validation of the Software

#### Reconstruction Algorithm Verification

To verify the accuracy of the reconstruction algorithm, we compared the dose calculated by the TPS with the reconstructed 2D dose distribution with the same beam parameters. The mean local dose difference was calculated for the proton and carbon ion plans. Ten carbon ion plans with 25 beams and ten proton plans with 25 beams were used for algorithm verification. For each beam, comparisons were performed at three different depths, one was at the proximal edge of the high dose region, another was at the middle of the high dose region, and the third was at the distal end of high dose region. All dose reconstructions used 1-mm grid size. The doses calculated by TPS with 2- or 3-mm grid sizes were interpolated to a 1-mm grid size.

#### Sensitivity of Plan Delivery Accuracy to Different Deviations

To study the sensitivity of plan delivery accuracy to spot size deviation, position deviation, and particle number deviation. Different levels of Gaussian-like random errors were manually added to the PBP plan parameters to generate modified plans. Then, the reconstructed dose based on modified plans was compared with the original plan dose calculated by the TPS. During simulation, the spot size deviation levels ranged from 1% to 34%, the spot position deviation levels ranged from 0.1 to 3 mm, and the particle number deviation levels ranged from 0.1% to 2%. Deviation in spot size, spot position, and particle number was investigated separately. The sigma value of the Gaussian-like random error is obtained through the comparison of beam parameters between the PBP and the PBR of the 20 plans. The standard deviation of the spot size difference, spot position difference, and particle number difference was used as the standard deviation in a Gaussian distribution to generate random errors. The dose comparison for each beam was performed at three different depths, which were the same as the depth used for algorithm verification. Each deviation level was calculated 10 times for each beam to maintain the data stability. A gamma pass rate with 3 mm/3% criteria was applied to evaluate the difference between the original plan dose and modified plan dose for carbon and proton beams, respectively.

#### Plan Delivery Verification Using Homemade Software

Finally, 52 treatment plans were retrospectively analyzed using homemade software for clinical validation. In which there were 25 carbon ion plans with 52 beams (7 plans for head and neck tumor, 8 plans for lung tumor, and 10 plans for abdomen tumor) and 27 proton plans with 52 beams (10 plans for head and neck tumor, 8 plans for lung tumor, and 9 plans for abdomen tumor). Three different depths were chosen for each beam to do the dose comparison. Spot size deviation in the x and y directions (ΔFx, ΔFy), position deviation in the x and y directions (Δx, Δy), and particle number deviation in each spot (ΔN) were also analyzed for each pencil beam.

**Figure 2:**
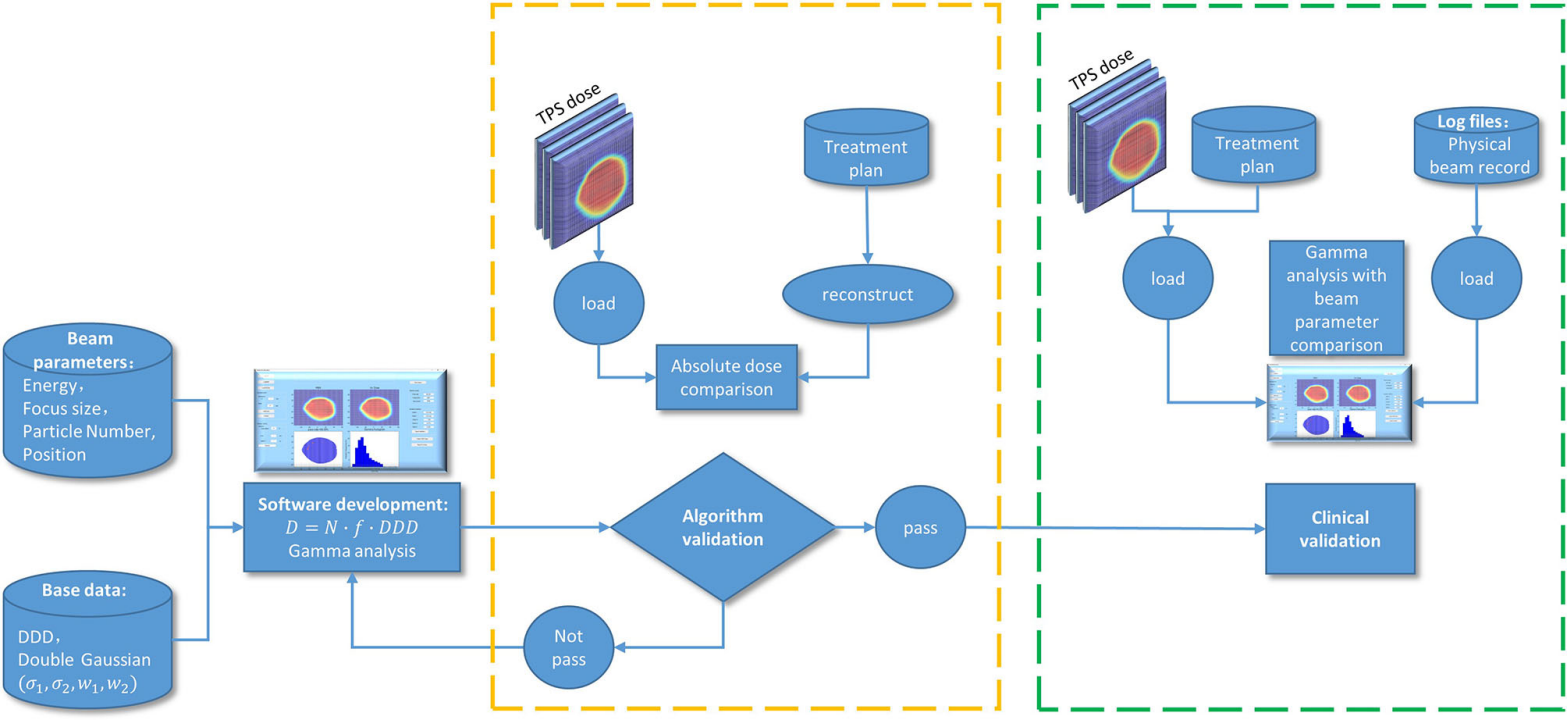
A flow diagram of the program development, technical validation, and clinical validation.

To sum up, the process of the program development, technical validation, and clinical validation is shown in **Figure 2**.

## Results

**Figure 3:**
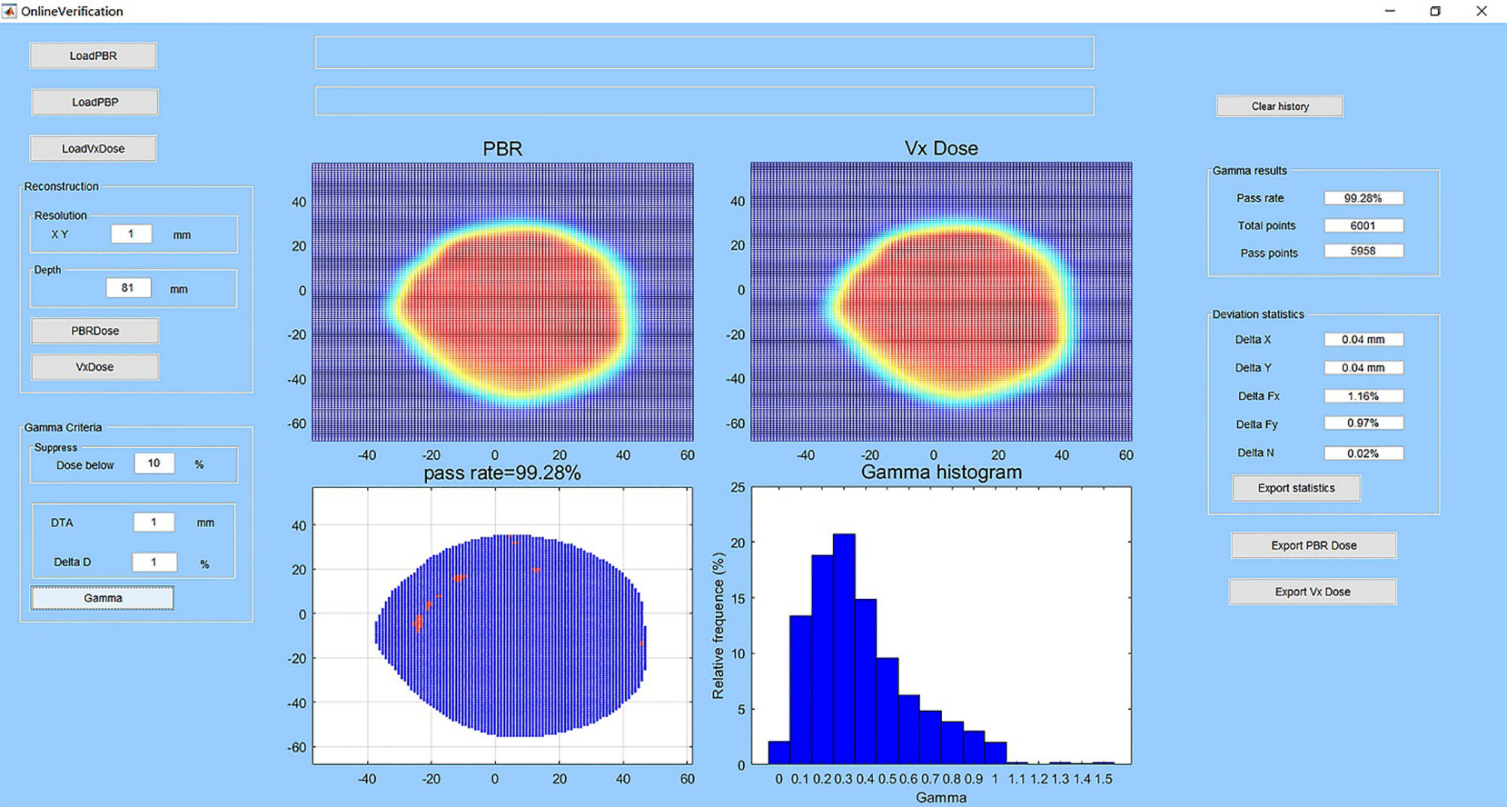
User interface of the homemade software and an example of verification. The subplot entitled “PBR” is the reconstructed dose in the water-phantom at the selected depth. The subplot entitled “Vx Dose” is the dose calculated by the TPS imported to the software. The subplot entitled “pass rate” shows the gamma pass rate, and spots that do not pass the gamma criteria are in red color. The subplot entitled “gamma histogram” shows the gamma index distribution. At left are the buttons for data import and dose reconstruction and the edit boxes for setting dose reconstruction and gamma analysis parameters. At right are the edit boxes for analysis results and buttons for data export.

The user interface of homemade software and an example of verification are shown in **Figure 3**. The software has dose reconstruction and gamma analysis functions. Users can manually set the reconstruction resolution and depth in water and change the gamma analysis criteria. The reconstructed dose distribution can be exported in DICOM format. The software can also calculate the deviations of spot size, spot position, and particle number for each beam between the actual delivered value and planned value. These statistics can also be exported for further analysis. For the reconstruction of one dose slice, it takes approximately 10 to 40 s in a personal laptop.

For the verification of the dose reconstruction algorithm, the mean dose difference between the dose calculated by the TPS and the dose reconstructed from the same parameters are 0.70% ± 0.24% and 0.51% ± 0.25% for carbon ion beam and proton beam, respectively. This small difference may be caused by the data interpolation of the depth dose distribution during dose reconstruction and interpolation of the dose grid size.

**Figure 4:**
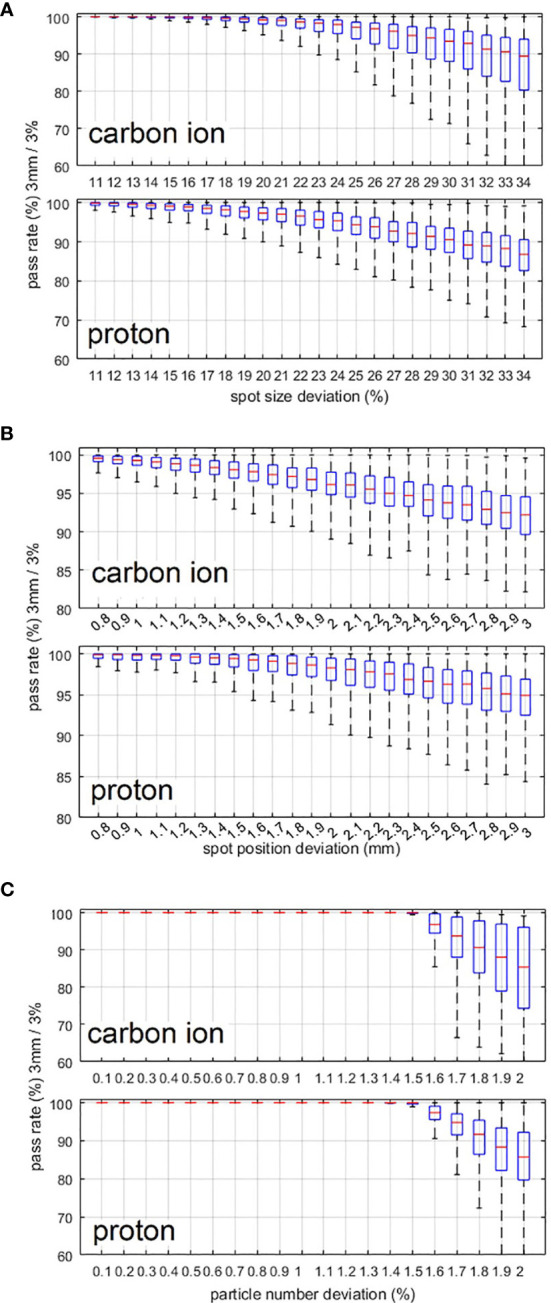
**(A–C)** Variation in the gamma pass rate with changes in the spot size deviation level, spot position deviation level, and particle number deviation level. 3 mm/3% criterion was used in the gamma analysis.

For the sensitivity investigation, **Figure 4** shows the relationship between the gamma pass rate and the mean deviation level of the spot size, spot position, and particle number. From **Figure 4A**, we can see that proton beams are more sensitive to spot size deviation. From **Figures 4B, C**, we can see that carbon ion beams are more sensitive to spot position deviation and particle number deviation. To achieve a 90% gamma pass rate with 3 mm/3% criteria, the average spot size deviation, position deviation, and particle number deviation should be within 23%, 1.9 mm, and 1.5% and 20%, 2.1 mm, and 1.6% for the carbon ion beam and proton beam, respectively.

For the actual plan delivery verification results of the 52 treatment plans using homemade software, the gamma pass rate of the comparison between the dose reconstructed from the PBR and the dose calculated by the TPS is shown in **Table 1**. The deviations of spot size, spot position, and particle number between the actual delivered value (PBR) and planned value are also listed in the table. Local dose normalization was used in gamma evaluation for an area with dose above 10% of the maximum dose. The gamma pass rates for carbon ion beams and proton beams are about 99% and 98%, respectively, which is consistent with the simulation results in **Figure 4**.

**Table 1:** Plan delivery verification results of carbon ion and proton plans using homemade software.

		carbon ion	*p* (carbon ion)	proton	*p* (proton)
**Gamma pass rate (1 mm/1%)**	Proximal edge of high dose region	98.93% ± 0.74%		97.80% ± 2.01%	
	Middle of high dose region	98.91% ± 0.51%		97.84% ± 1.49%	
	Distal end of high dose region	99.09% ± 0.65%		98.19% ± 1.92%	
	ΔFx (%)	4.33 ± 2.64		5.89 ± 2.71	
	ΔFy (%)	6.35 ± 3.28		4.08 ± 1.79	
	$\sqrt{\Delta Fx^{2}+\Delta Fy^{2}}$	7.97 ± 3.65	0.129	7.46 ± 2.50	0.09
	Δx (mm)	0.05 ± 0.02		0.04 ± 0.03	
	Δy (mm)	0.03 ± 0.01		0.04 ± 0.02	
	$\sqrt{\Delta x^{2}+\Delta y^{2}}$	0.06 ± 0.02	0.065	0.06 ± 0.03	0.836
	ΔN (%)	0.02 ± 0.01	0.325	0.01 ± 0.01	0.628

The data shown represents the mean value with 1 s.d. The relationship of the gamma pass rate and different deviations is analyzed using multiple liner regression.

*ΔFx and ΔFy are the spot size deviations in the x and y directions, respectively. Δx and Δy are the position deviations in the x and y directions. ΔN is the particle number deviation in each spot. A p<0.05 indicates that the pass rate decreases when deviation increases.

## Discussion

At present, all plan verification systems for proton and carbon ion radiotherapy are designed for pretreatment verification, which cannot provide plan delivery information during treatment. Although all radiotherapy facilities have beam application and monitoring systems, they cannot provide quantitative dose information. It is very important to perform actual treatment verification to quantitatively show the dose differences between the actual dose delivered and the dose calculated by the TPS. In this study, we introduced a method and developed verification software based on log files for scanning beam proton and carbon ion radiotherapy, which considered the impacts of spot size deviation, spot position deviation and particle number deviation. The algorithm for dose reconstruction was validated by the good consistency between the dose calculated by the TPS and the dose reconstructed from the same beam parameters. In the future, we will try to design some methods to do actual planar dose measurement in water to further verify the reconstruction algorithm. Several studies have using similar method to do the quality assurance for proton therapy (21–24). The position deviation and MU deviation of pencil beams were analyzed for dose difference analysis. However, the spot size deviation of the pencil beam was not analyzed in these studies, which is also a non-negligible reason affecting treatment accuracy. They did not analyze the sensitivity of plan verification results to different beam parameter errors, which are useful for the plan design and system setting of proton and carbon ion radiotherapy.

The relationship between plan delivery accuracy and different deviation levels was also investigated. As a result of the smaller spot size of the carbon ion beam than that of the proton beam, the pass rate of carbon ion beams is more sensitive to spot position deviation and particle number deviation, and the pass rate of the proton beam is more sensitive to spot size deviation. **Figure 4A** shows that proton beams are more sensitive to spot size deviation, which is due to the larger spot sizes of proton beams than that of carbon ion beams. First, for larger spot sizes the spot size deviation of one spot will affect more areas. Second, a larger spot size will result in a sparse spot distribution, which can lead to less mutual compensation between spots especially for small or narrow areas. **Figures 4B, C** show that carbon ion beams are more sensitive to spot position deviation and particle number deviation, because the spot size of carbon ion beams is smaller than that of proton beams. It is obvious that for smaller spot sizes, the same particle number deviation and absolute spot position deviation will result in a larger-dose deviation in the local area.

For actual treatment verification using homemade software, **Table 1** reveals that the gamma pass rates are very high for both proton and carbon ion beams at present deviation level. A limitation of this online verification method and homemade software is that the beam energy cannot be verified. However, monthly quality assurance of beam energy using peak-finder and precise parameter control of bending magnet and extraction system can ensure the energy accuracy. In addition, routine plan verification using water tank and ionization chambers can also ensure the accuracy of energy. We can see that the actual plan verification results in **Table 1**, and simulation results in **Figure 4** are consistent. In general, verification using homemade software reveals errors during patient treatment, which means that our methods and software are feasible and reliable for plan delivery verification. Although the beam parameters are measured in the beam nozzle, they are converted to values at isocenter by a lookup table and stored in the log file. To ensure the accuracy of BAMS detectors and the look-up table, routine quality assurance is carried out daily and weekly. During daily QA, the beam position and spot size of a set of energies are checked by film. The transmission ion chambers in the BAMS are also calibrated by a farmer chamber every day. The lookup table to convert the values measured in BAMS to values at the isocenter is checked every week using film and MWPC.

In this study, each plan contains multi energies and all the energies contribute to the dose distribution more or less. Currently, we cannot separate each beam plan based on the energy slice and measure each energy’s contribution to the total plan dose. What is more, most of the plans are created based on intensity-modulated proton/carbon therapy method, it is difficult to tell any proton energy dependence. During the verification measurement with water phantom, the plan which contains low energies sometimes may fail during the measurement, and these plans usually contain range shifter. Lots of studies had revealed that using pencil beam algorithm to calculate the plan with range shifter can exist bigger error (25, 26). For the plan without range shifter but using lower energy, the lower energies have less contribution to the plan dose. Comparing each iso-energy slice can give us more information, but just set the criteria based on single energy maybe too tight. How to consider each energy’s contribution and the dose deviation will be considered in the future.

Other than the analysis of the reconstructed dose distribution, the homemade software calculated the discrepancies of spot size, spot position, and particle number. These results could be part of the daily performance check of the beam delivery system during patient treatment and can help to analyze the results of gamma evaluation. In the future, we will integrate homemade software into the radiation oncology system, which will allow immediate analysis instantaneously after the beam is irradiated. The results could be part of the treatment report for each patient.

## Conclusions

We introduced a method and developed software to perform plan delivery verification based on log files for proton and carbon ion beam radiotherapy with a pencil-beam scanning technique. The plan delivery accuracy of carbon ion beam is more sensitive to spot position deviation and particle number deviation, and the plan delivery accuracy of the proton beam is more sensitive to spot size deviation. Clinical validation results show that the homemade software for treatment dose verification is feasible and reliable. Such a method can check the online performance of proton and carbon ion delivery systems and evaluate the impacts of beam parameter variations on the plan delivery accuracy, which will enhance current treatment verification processes.

## Data Availability Statement

The raw data supporting the conclusions of this article will be made available by the authors, without undue reservation.

## Author Contributions

JZ designed the study and drafted the manuscript. ZC wrote the program code. XW, YX, and YL participated in the data collection and analysis. All authors contributed to the article and approved the submitted version.

## Funding

This study was supported by research funding from the National Natural Science Foundation of China (11905035).

## Conflict of Interest

The authors declare that the research was conducted in the absence of any commercial or financial relationships that could be construed as a potential conflict of interest.
